# Acetylcholinesterase Activity, Cohabitation with Floricultural Workers, and Blood Pressure in Ecuadorian Children

**DOI:** 10.1289/ehp.1205431

**Published:** 2013-01-25

**Authors:** Jose R. Suarez-Lopez, David R. Jacobs, John H. Himes, Bruce H. Alexander

**Affiliations:** 1 Division of Epidemiology and Community Health, University of Minnesota, Minneapolis, Minnesota, USA; 2 Fundación Cimas del Ecuador, Quito, Ecuador; 3 Department of Nutrition, School of Medicine, University of Oslo, Oslo, Norway; 4 Division of Environmental Health Sciences, University of Minnesota, Minneapolis, Minnesota, USA

**Keywords:** acetylcholinesterase, AChE, agricultural communities, agriculture, blood pressure, children, Ecuador, epidemiology, pesticides, secondary exposure

## Abstract

Background: Acetylcholinesterase (AChE) inhibitors are commonly used pesticides that can effect hemodynamic changes through increased cholinergic stimulation. Children of agricultural workers are likely to have paraoccupational exposures to pesticides, but the potential physiological impact of such exposures is unclear.

Objectives: We investigated whether secondary pesticide exposures were associated with blood pressure and heart rate among children living in agricultural Ecuadorian communities.

Methods: This cross-sectional study included 271 children 4–9 years of age [51% cohabited with one or more flower plantation workers (mean duration, 5.2 years)]. Erythrocyte AChE activity was measured using the EQM Test-mate system. Linear regression models were used to estimate associations of systolic blood pressure (SBP), diastolic blood pressure (DBP), and heart rate with AChE activity, living with flower workers, duration of cohabitation with a flower worker, number of flower workers in the child’s home, and number of practices that might increase children’s exposure to pesticides.

Results: Mean (± SD) AChE activity was 3.14 ± 0.49 U/mL. A 1-U/mL decrease in AChE activity was associated with a 2.86-mmHg decrease in SBP (95% CI: –5.20, –0.53) and a 2.89-mmHg decrease in DBP (95% CI: –5.00, –0.78), after adjustment for potential confounders. Children living with flower workers had lower SBP (–1.72 mmHg; 95% CI: –3.53, 0.08) than other children, and practices that might increase exposure also were associated with lower SBP. No significant associations were found between exposures and heart rate.

Conclusions: Our findings suggest that subclinical secondary exposures to pesticides may affect vascular reactivity in children. Additional research is needed to confirm these findings.

Cholinesterase inhibitors such as organophosphate and carbamate insecticides are commonly used pesticides in agriculture worldwide that can increase parasympathetic activity via inhibition of acetylcholinesterase (AChE) activity. Alterations in the cholinergic system can have varying effects on blood pressure regulation depending on whether nicotinic or muscarinic stimulation by acetylcholine predominates. Acetylcholine can lower blood pressure via vasodilation through nitric oxide, cyclooxygenase, and endothelium-derived hyperpolarizing factor pathways (de Wit et al. 1999; Hatoum et al. 2003a, 2003b; Leung et al. 2006) through the stimulation of muscarinic M3 receptors in vascular endothelium (Beny et al. 2008; Eltze et al. 1993; Gericke et al. 2011; Lamping et al. 2004). However, acetylcholine can also increase blood pressure by stimulating nicotinic receptors in the sympathetic system (Claassen et al. 2009), as observed among adults with orthostatic hypotension in response to cholinesterase inhibitor drugs (Gales and Gales 2007; Singer et al. 2003). Currently, it is unclear whether alterations in AChE activity from exposure to cholinesterase inhibitors can affect blood pressure.

Children of agricultural workers are at risk of pesticide contamination from secondary routes of exposure, including pesticide drift due to residential proximity to plantations, and from pesticides inadvertently carried into homes by workers, for example, on their clothes or skin. (Coronado et al. 2011; Curl et al. 2002; Fenske et al. 2002; Gladen et al. 1998; Lambert et al. 2005; Lu et al. 2000; McCauley et al. 2001; Simcox et al. 1995; Suarez-Lopez et al. 2012). In general, these secondary pesticide exposures are too small to elicit overt clinical manifestations, but they may persist throughout the duration of the child’s cohabitation with the agricultural worker (which may last many years).

We examined whether lower AChE activity from secondary pesticide exposure and cohabitation with a flower plantation worker (flower worker) were associated with changes in blood pressure and heart rate among children living in agricultural communities in Ecuador, where there is an active fresh-cut flower industry. The floricultural industry in Ecuador regularly uses various types of pesticides, including cholinesterase inhibitors such as organophosphate and carbamate insecticides (Grandjean et al. 2006; Harari 2004), in addition to pyrethroid insecticides and various fungicides and herbicides.

## Methods

*Study description*. The Secondary Exposure to Pesticides among Infants, Children and Adolescents Study (ESPINA: Estudio de la Exposición Secundaria a Plaguicidas en Infantes, Niños y Adolescentes) is a study of children living in Pedro Moncayo County, Pichincha, Ecuador. The county has substantial floricultural activity with an approximate production area of 1,800 hectares (5.3% of the county’s surface area) (Gobierno Municipal del Canton Pedro Moncayo 2011). The industry employs approximately 21% of adults in the county (Suarez-Lopez et al. 2012).

In 2008, the ESPINA study examined 313 children 4–9 years of age, including a subset who participated in the 2004 Survey of Access and Demand of Health Services in Pedro Moncayo County conducted by Fundacion Cimas del Ecuador (73%), as well as new volunteers (27%) (Suarez-Lopez et al. 2012). We conducted in-person home interviews of 451 adults who lived with the examined children. Additional participant recruitment information is described elsewhere (Suarez-Lopez et al. 2012). For these cross-sectional analyses, we included 271 (88%) children who had information on all covariates of interest.

Informed consent, parental authorization of child participation, and child assent of participants ≥ 7 years of age was provided for all study participants. This study was approved by the institutional review boards of Fundacion Cimas del Ecuador and the University of Minnesota.

*Measures.* In-person home interviews of children’s parents and other adults were used to obtain information on socioeconomic status, demographics, health, and direct and indirect pesticide exposures of household members. Children’s height was measured using a height board, following recommendations by the World Health Organization (WHO 2008), and weight (standing) was measured using a digital scale (Tanita model 0108MC; Corporation of America, Arlington Heights, IL, USA). Resting heart rate was measured by a 30-sec auscultation, before blood pressure measurement. Systolic blood pressure (SBP) and diastolic blood pressure (DBP) were measured with a pediatric aneroid sphygmomanometer (model BF683W; Omron Healthcare Inc., Lake Forest, IL, USA), following protocols recommended by the American Heart Association (Pickering et al. 2005). Measurements were taken after 3–5 min of rest. Children were in the seated position with the antecubital fossa supported at heart level, with uncrossed legs and both feet on the floor. Blood pressure was measured twice, and averages of the two SBP and DBP values were used in analyses.

Erythrocytic AChE activity and hemoglobin concentration were measured from a single finger stick sample using the EQM Test-mate ChE Cholinesterase Test System 400, AChE Erythrocyte Cholinesterase Assay Kit 470 (EQM, Cincinnati, OH, USA).

The distance from each child’s residence to the nearest flower plantation was calculated using ArcGIS 9.3 (ESRI, Redlands, CA, USA) based on geographical coordinates obtained from portable global positioning system (GPS) receivers. Additional details regarding the GPS data collection and calculations are described elsewhere (Suarez-Lopez et al. 2012).

*Statistical analysis*. We used multiple linear regression to estimate associations between flower worker cohabitation (yes or no), duration of flower worker cohabitation (continuous), and AChE activity (continuous) with SBP, DBP (including standardized values for SBP and DBP), and heart rate. Statistical significance was defined using an α of 0.05. In addition we plotted the association of blood pressure as a linear function of AChE and adjusted least square mean estimates for SBP and DBP according to octiles of AChE activity to visually assess model fit.

The number of flower workers concurrently living at the child’s home was modeled as a continuous variable and as an ordinal variable (0 = none, 1 = 1 flower worker, 2 = ≥ 2 flower workers).

We obtained information about practices among flower workers that would likely increase the amounts of pesticides inadvertently brought home, including how often they washed their hands or showered before leaving work, how often they took their work shoes home, and how often they wore their work clothes home, with four possible responses: never, 1–2 days/week, 3–4 days/week, or always. In addition, they were asked where they washed their work clothes (at work, at home, or other), and whether they took tools from work to their home (yes or no).

We previously found that the most common sources of pesticide introduction into the home were washing work clothes at home (95%), never showering at work (45%), infrequent (1–2 days/week) removal of work clothes before leaving work (37%), and bringing work shoes (18%) and tools (16%) home; additional information is described elsewhere (Suarez-Lopez et al. 2012).

A household-level value of flower worker practices was assigned to each child. When there was more than one flower worker living in a household, we counted only the worst practice value among all such workers. We then derived a variable indicating the total number of “bad practices” (likely to result in home contamination with pesticides), which were defined as the two worst practices in questions with four options (e.g., bringing work clothes home always or 3–4 times/week were counted as one “bad practice”) and as the worst practice in all other questions (e.g., washing work clothes at home), with possible values of 0–6. Children of nonagricultural workers were assigned a value of 0. We previously found that the number of bad practices by flower workers was associated with lower AChE activity in children (Suarez-Lopez et al. 2012).

Height-for-age and BMI (body mass index)–for-age *z*-scores were calculated using WHO growth standards (WHO Multicentre Growth Reference Study Group 2006). Standardized blood pressure (*z*-scores), appropriate for age, sex, and height-for-age, were calculated using equations from the *Fourth Report on the Diagnosis, Evaluation, and Treatment of High Blood Pressure in Children and Adolescents* (National High Blood Pressure Education Program Working Group on High Blood Pressure in Children and Adolescents 2005).

For each predictor (AChE activity, cohabitation with a flower worker, duration of cohabitation with a flower worker, number of flower workers in the home, or number of “bad practices”), we used two models to estimate associations with SBP, DBP, or heart rate. We examined children during July and August, roughly 2–3 months after a period of increased pesticide use related to the Mother’s Day (May) surge of flower production. Because AChE activity can reflect exposures to organophosphates within the previous 82 days (Mason 2000), children examined earlier in July may have had lower AChE activity than children examined in August. Therefore, model 1 was adjusted for examination date, in addition to age, sex, race [defined by parents: mestizo (e.g., mix of indigenous and white, mestizo with mestizo), indigenous, black, or white], and height-for-age *z*-score. When the predictor was AChE activity, hemoglobin concentration was added to model 1. In addition to model 1 covariates, model 2 was adjusted for heart rate, income, number of smokers living at home, distance from the residence to the nearest flower plantation edge, pesticide use within the household or on the household lot, and pesticide use by contiguous neighbors. When the predictor was AChE activity, flower worker cohabitation was added to model 2. When heart rate was the outcome of interest, model 2 did not include heart rate as a covariate. We tested the associations for effect modification by sex and race through the addition of an interaction term between each predictor and either sex or race in the model, using an α cut-off of 0.05. Data were analyzed with SAS (version 9.2; SAS Institute Inc., Cary, NC, USA).

## Results

*Participant characteristics*. Children who lived with a flower worker had similar distributions of age, sex, income, and number of smokers at home compared with children who lived with nonagricultural workers, but were more likely to be classified as indigenous and to live closer to flower plantations (Table 1). On average, all children in the study population were short for their age when compared with the WHO normative sample, with an overall height-for-age *z*-score of –1.26 SDs. Forty-nine percent of participants did not live with flower workers, whereas 28% lived with one flower worker and 23% lived with two or more. The average number of flower workers in the homes of children who lived with flower workers was 1.64. Among children with information on bad practice scores (*n* = 228, 84%), 116 (51%) had 0 bad practices (including 115 children who did not live with flower workers), whereas 17% had 1–2, 19% had 3, and 13% had 4–6 bad practices.

**Table 1:** Participant characteristics.

Participant characteristics	All participants (n = 271)	Cohabitation with flower plantation worker (n = 138; 51%)	Cohabitation with nonagricultural worker (n = 133; 49%)	p-Value
Demographic and socioeconomic status				
Age (years)	6.6 ± 1.6	6.4 ± 1.6	6.8 ± 1.6	0.02
Male sex	51	51	52	0.85
Race
Mestizo	76	68	84	0.002
Indigenous	23	30	14	0.01
Other	1	1	2	0.66
Monthly incomea	3.1 ± 0.8	3.1 ± 0.7	3.1 ± 1.0	0.97
No. of smokers at home	0.26 ± 0.46	0.25 ± 0.45	0.27 ± 0.46	0.66
Residence distance to nearest flower plantation (m)	347 (186–602)	326 (170–610)	432 (208–602)	0.55
Duration of flower worker cohabitation (years)		5.2 ± 2.0		—
No. of flower workers at home		1.64 ± 1.1	0	—
No. of “bad practices”b		2.9 ± 0.9	0	—
Anthropometric and blood measurements
Height (cm)	112.1 ± 10.3	110.2 ± 10.2	114.1 ± 10.2	0.002
Height-for-age z-score	–1.26 ± 0.96	–1.41 ± 1.04	–1.10 ± 0.85	0.01
BMI (kg/m2)	16.1 ± 1.4	16.1 ± 1.1	16.2 ± 1.6	0.48
BMI-for-age z-score	0.34 ± 0.8	0.35 ± 0.71	0.32 ± 0.88	0.77
Systolic blood pressure (mmHg)	93.2 ± 8.3	92.2 ± 7.6	94.2 ± 8.9	0.05
Systolic blood pressure (z-score)	–0.03 ± 0.72	–0.06 ± 0.67	0.00 ± 0.77	0.45
Diastolic blood pressure (mmHg)	49.4 ± 7.3	49.3 ± 7.6	49.6 ± 7.0	0.76
Diastolic blood pressure (z-score)	–0.50 ± 0.62	–0.47 ± 0.66	–0.52 ± 0.56	0.53
Heart rate (beats/min)	85.1 ± 12.4	86.4 ± 12.4	83.8 ± 12.4	0.08
Acetylcholinesterase (U/mL)	3.14 ± 0.49	3.08 ± 0.50	3.20 ± 0.47	0.05
Hemoglobin (g/dL)	12.6 ± 1.2	12.5 ± 1.3	12.7 ± 1.0	0.15
Values are mean ± SD, percent, or median (25th–75th percentile). aMonthly income categories (US$): 1 = 0–50, 2 = 51–150, 3 = 151–300, 4 = 301–500, 5 = 501–1,000, 6 = > 1,000. bn = 228.				

**Figure 1:**
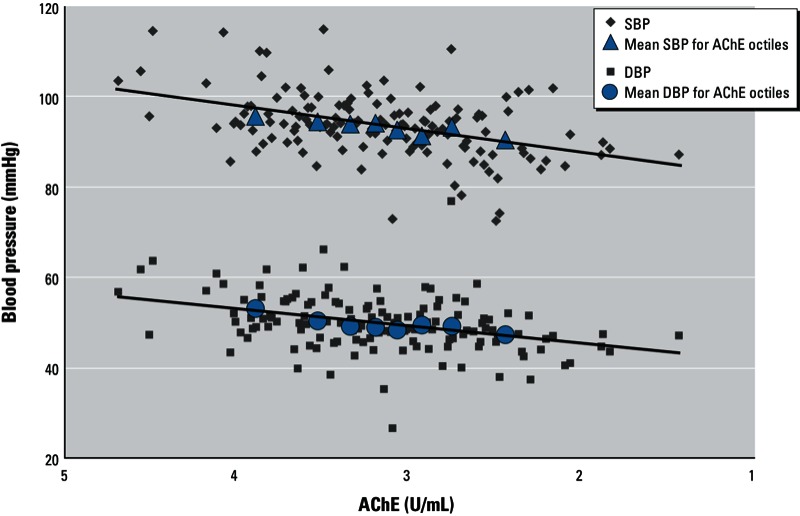
Adjusted associations between AChE activity and blood pressure (*n* = 271). The regression line was estimated from adjusted linear regression models for each outcome. All models were adjusted for age, sex, race, height-for-age z-score, heart rate, hemoglobin concentration, income, number of smokers living at home, residence distance to nearest flower plantation edge, pesticide use within household lot, pesticide use by contiguous neighbors, and examination date.

*AChE activity and blood pressure*. AChE activity was positively associated with blood pressure in both models (Table 2). In model 2, every 1-U/mL decrease in AChE activity was associated with a 2.86-mmHg decrease in mean SBP (95% CI: –5.20, –0.53) and a 2.89-mmHg decrease in mean DBP (95% CI: –5.00, –0.78). Adjusted least square means for SBP and DBP estimated for each octile of AChE activity were closely aligned with the estimated linear trends, thus providing visual evidence of good model fits (Figure 1). In model 2, each 1-U/mL decrease in AChE activity was associated with decreases in *z*-scores for SBP and DBP of –0.26 SD (95% CI: –0.48, –0.04) and –0.22 SD (95% CI: –0.40, –0.03), respectively.

**Table 2:** Adjusted associations between measures of secondary pesticide exposure and blood pressure and heart rate differences among children (*n* = 271).

Predictor	Model 1 SBP [mmHg (95% CI)]	Model 1 DBP [mmHg (95% CI)]	Model 1 heart rate [BPM (95% CI)]	Model 2 SBP [mmHg (95% CI)]	Model 2 DBP [mmHg (95% CI)]	Model 2 heart rate [BPM (95% CI)]
AChE (per 1-U/mL decrease)	–2.20 (–4.51, 0.11)**	–3.19 (–5.21, –1.17)*	–2.24 (–5.90, 1.41)	–2.86 (–5.20, –0.53)*	–2.89 (–5.00, –0.78)*	–3.01 (–6.79, 0.77)
Flower worker cohabitation (yes vs. no)	–1.50 (–3.34, 0.33)	–0.43 (–2.06, 1.20)	1.91 (–0.99, 4.80)	–1.72 (–3.53, 0.08)**	–0.49 (–2.12, 1.14)	1.62 (–1.27, 4.52)
Duration of cohabitation with a flower worker (per year)	–0.33 (–0.64, –0.02)*	–0.13 (–0.41, 0.15)	0.12 (–0.37, 0.61)	–0.32 (–0.64, –0.02)*	–0.12 (–0.39, 0.16)	0.11 (–0.38, 0.61)
No. of flower workers at home (per worker)	–0.68 (–1.49, 0.13)	–0.36 (–1.08, 0.36)	0.34 (–0.94, 1.62)	–0.79 (–1.58, 0.005)#	–0.43 (–1.14, 0.28)	0.51 (–0.76, 1.79)
No. of bad practices (per practice)a	–0.66 (–1.31, –0.01)*	–0.34 (–0. 90, 0.22)	0.63 (–0.35, 1.62)	–0.82 (–1.45, –0.20)*	–0.37 (–0.92, 0.18)	0.56 (–0.43, 1.56)
BPM, beats per minute. Model 1: age, sex, race, height-for-age z-score and examination date. When the predictor was AChE activity, hemoglobin concentration was also included in the model. Model 2: model 1 + heart rate, income, number of smokers living at home, residence distance to nearest flower plantation edge, pesticide use within household lot, and pesticide use by contiguous neighbors. When heart rate was the outcome of interest, model 2 did not include heart rate as an adjustment variable; when the predictor was AChE activity, flower worker cohabitation was added to the model. an = 228. *p < 0.05. **p = 0.06. #p = 0.052.						

AChE activity was positively associated with resting heart rate, but estimated decreases in mean values were not statistically significant (Table 2).

*Flower worker cohabitation and blood pressure*. Children living with flower workers had borderline lower SBP in model 2 compared with children living with nonagricultural workers. Duration of cohabitation with a flower worker and number of flower workers in the home were associated with lower mean SBP and DBP, though estimated differences in mean DBP were smaller and nonsignificant (Table 2). When analyzing number of flower workers as an ordinal variable, compared with children who did not live with flower workers, mean SBP among those who lived with one flower worker or with two or more flower workers decreased by 1.20 mmHg (95% CI: –3.31, 0.92) and 2.56 mmHg (95% CI: –4.80, –0.32), respectively. Further adjustment for AChE activity and hemoglobin concentration weakened associations between SBP and cohabitation with a flower worker (–1.59 mmHg; 95% CI: –3.37, 0.19 compared with –1.72 mmHg; 95% CI: –3.53, 0.08 for model 2), with duration of cohabitation (–0.27 mmHg per year of cohabitation; 95% CI: –0.58, 0.03 compared with –0.32; 95% CI: –0.64, –0.02), and with the number of flower workers living at home (–0.71; 95% CI: –1.49, 0.08 compared with –0.79; 95% CI: –1.58, 0.005). Adjustment for BMI-for-age had negligible effects on the association (results not shown). In model 2, every “bad practice” performed by flower workers was associated with lower SBP (mean –0.82 mmHg; 95% CI: –1.45, –0.20) and lower DBP (mean –0.37 mmHg; 95% CI: –0.92, 0.18).

Lower mean *z*-scores of SBP but not DBP were associated in model 2 with cohabitation with a flower worker versus no cohabitation (SBP: –0.17 SD; 95% CI: –0.34, –0.003; DBP: –0.06 SD; 95% CI: –0.20, 0.08), duration of cohabitation with a flower worker (SBP: –0.03 SD per year; 95% CI: –0.06, –0.004; DBP: –0.01 SD per year; 95% CI: –0.04, 0.01), number of flower workers (SBP: –0.07 SD; 95% CI: –0.15, 0.000; DBP: –0.04 SD; 95% CI: –0.10, 0.02), and number of bad practices (SBP: –0.04 SD; 95% CI: –0.08, 0.01).

There was effect modification by race in the association of flower worker cohabitation (*p*-interaction = 0.002), duration of cohabitation (*p*-interaction = 0.03), and number of flower workers (*p*-interaction = 0.03) with DBP. The associations with DBP were stronger among children classified as indigenous compared with mestizo for the following predictors: flower worker cohabitation status (indigenous: –4.45 mmHg; 95% CI: –8.51, –0.39; mestizo: –0.75 mmHg; 95% CI: –2.60, 1.09), duration of cohabitation (indigenous: –0.26 mmHg; 95% CI: –1.00, 0.49; mestizo: 0.01 mmHg; 95% CI: –0.31, 0.33), and number of flower workers living with child (indigenous: –1.00 mmHg; 95% CI: –2.09, 0.09; mestizo: 0.25 mmHg; 95% CI: –0.73, 1.23). No other interaction examined (i.e., between race or sex and other exposures in relation to SBP or DBP) differed significantly between indigenous and mestizo children or between boys and girls (data not shown).

Resting heart rate was positively but not significantly associated with flower worker cohabitation (Table 2).

## Discussion

AChE activity was associated with SBP and DBP even after adjustment for resting heart rate, thus suggesting that subclinical pesticide exposures may influence vascular reactivity among children.

The negative associations of cohabitation with a flower worker and longer duration of cohabitation with SBP suggest that the amount of take-home pesticide exposure by flower workers may be sufficient to induce physiological changes in children. Additional evidence in support of this hypothesis includes the negative associations of SBP with the number of flower workers living at home and the number of “bad practices” that may increase the likelihood of take-home pesticide exposures by flower workers. In this population, 95% of flower workers washed their work clothes at home, 45% never showered at work before going home, and many regularly brought work clothes and tools home (Suarez-Lopez et al. 2012). The substantial number of “bad practices” among flower workers in our study suggests that efforts by the industry to implement work environments and effective education to reduce take-home pesticide exposures are insufficient. Relatively easy interventions—such as continuing education for flower workers and their families and providing laundry services for work clothes at the plantations—could yield a substantial decrease in secondary pesticide exposures to families of agricultural workers and could benefit the development of a significant number of children living in agricultural communities.

Although we did observe a statistically significant interaction with race in the associations of flower worker cohabitation, duration of cohabitation, and number of flower workers living with DBP, we do not believe this association is important. With complementary and related information, these three exposure variables represent one exposure construct: cohabitation with a flower worker. Given the genetic similarities between indigenous and mestizos and the fact that there was evidence of effect modification of, in essence, one exposure–outcome relation, it is likely that the observed difference by race may be spurious.

The estimated association of cohabitation with a flower worker and blood pressure may be conservative because participants were examined in July and August, which are months of decreased flower production.

The results of this investigation suggest a physiological disturbance associated with cohabitation with agricultural workers. This concept is corroborated by a previous finding in this study population, where children living with flower workers had lower AChE activity compared with nonagricultural workers, and lower AChE was associated with longer duration of cohabitation and a greater number of bad practices (Suarez-Lopez et al. 2012).

The estimated average blood pressure decreases were relatively small, but the amount of cholinesterase inhibitor pesticide exposure is also likely to be small due to the secondary nature of the exposure. Although lower blood pressure may be beneficial in many contexts, it is a concern that likely reflects physiological alterations of low-dose exposures to pesticides in children. Additionally, the long-term consequences of such effects on development are uncertain. However, our findings must be replicated in other populations before conclusions about causal effects can be made.

The pesticide exposure levels that children in Pedro Moncayo County are subjected to may not be very different from those of similar communities in the United States and other developed countries. A study of Ecuadorian children living in the vicinity of the present study area reported that urinary metabolite concentrations of organophosphate pesticides were similar to those of a representative sample of children in the United States (Grandjean et al. 2006). Furthermore, the mean AChE activity in our study population for children living with flower workers (3.08 U/mL) and agricultural workers (3.20 U/mL) were consistent with mean levels reported for Hispanic children living in agricultural (3.0 U/mL) and nonagricultural families (3.1 U/mL) in Oregon (USA) (Higgins et al. 2001). The higher AChE values reported in our study are likely attributable to the older age of our participants (4–9 years vs. 3–6 years). Among ESPINA study participants we observed that age was associated with an AChE increase of 0.05 U/mL per year of life (Suarez-Lopez et al. 2012). If we assume a 2.1-year mean age difference between the studies (Oregon study *≈* 4.5 years vs. ESPINA = 6.6 years), we obtain a correction of AChE activity of 0.1 U/mL, which is roughly the difference between the studies.

AChE activity is an appropriate method to assess past exposures to carbamate and organophosphate pesticides because it has a long recovery time, thus reflecting longer-term exposures than plasma cholinesterase (butyrylcholinesterase) or pesticide metabolite quantification (Lotti 1991; Mason 2000). Furthermore, AChE activity has a low intraindividual variability (Lefkowitz et al. 2007), in contrast with urinary metabolites of pesticides that have more intraindividual than interindividual variability (Griffith et al. 2011). AChE inhibition is a physiological response to the amount of pesticide exposure in relation to the individual’s sensitivity and ability to metabolize organophosphate pesticides; therefore, it can be interpreted without taking into consideration factors that affect organophosphate pesticide metabolism, such as paraoxonase activity. We could not calculate AChE inhibition relative to an unexposed baseline given the cross-sectional design of the study, and because most of our study population lived with a flower worker at some point in their lives, and many were born into a household of flower workers and were potentially exposed to pesticides *in utero*. Within the ESPINA study, we previously found inverse linear associations between cohabitation with a flower worker/duration of cohabitation and AChE activity based on a single measure, which suggests that the single measure is a valid indicator of pesticide exposure in children (Suarez-Lopez et al. 2012), considering that cohabitation with agricultural workers is associated with increased risk of pesticide exposures to family members (Curl et al. 2002; Fenske et al. 2002; Gladen et al. 1998; Lambert et al. 2005; Lu et al. 2000; McCauley et al. 2001; Simcox et al. 1995).

Children are particularly vulnerable to pesticide exposures due to behavior that fosters exposure (e.g., increased skin contact with floors/lawns from crawling or playing, hand-to-mouth behaviors) and physiological immaturity (i.e., increased skin surface and energy consumption for weight, increased breathing rates, decreased ability to detoxify pesticides, and sensitive developing organs) (Cohen Hubal et al. 2000; Faustman et al. 2000). Chronic, low-dose exposures to pesticides during childhood could result in physiological and developmental impairments, such as deficits in neurobehavior (Marks et al. 2010; Rohlman et al. 2005), and additional research is needed to determine long-term effects of such exposures.

The acetylcholine excess from AChE inhibition can increase the stimulation of both nicotinic and muscarinic receptors, thus producing systemic effects (Kwong 2002). Increased muscarinic receptor stimulation has been associated with increased salivation and lacrimation, nausea/vomiting, diarrhea, bradycardia, and vasodilation, whereas nicotinic receptor stimulation is mainly associated with muscular weakness/paralysis, hypertension, and tachycardia (Kwong 2002). Our finding that lower AChE activity was associated with lower blood pressure and a small, nonsignificant decrease in heart rate suggests that cholinesterase inhibitor pesticides may primarily induce vasodilation in children. We speculate that the blood pressure decreases may reflect a predominant stimulation of muscarinic M3 receptors in the vascular endothelium (Beny et al. 2008; Eltze et al. 1993; Gericke et al. 2011; Lamping et al. 2004).

Factors other than exposure to cholinesterase inhibitor pesticides may contribute to lower SBP among children who lived with a flower worker, given that associations persisted after adjusting for AChE activity. Agricultural workers are typically exposed to a mix of pesticides, and it is possible that non-cholinesterase inhibitor pesticides may also contribute to (or be responsible for) the decrease in SBP, because AChE inhibition could be acting as a proxy indicator of overall pesticide exposure.

Limitations of this study include the inability to establish temporality (given the cross-sectional study design) and the inability to evaluate individual pesticides or account for factors related to blood pressure such as prenatal toxicant exposures, concurrent and previous dietary patterns (e.g., salt and fat consumption), and perinatal health history.

The concomitant use of a variety of substances for stimulating plant growth and reducing pests by the floricultural industry (and agriculture in general) is a challenge when attempting to discern the impact of environmental pollutants on health. Our inability to measure different types of pesticides, growth stimulants or other pollutants is a limitation.

## Conclusions

Our findings suggest that subclinical exposures to cholinesterase inhibitor (and potentially other) pesticides, via take-home routes by flower workers, may induce physiological alterations in children resulting in decreased blood pressure. Generally, lower blood pressure has been associated with better cardiovascular outcomes, but the long-term cardiovascular effects of chronic low-dose pesticide exposures are unknown, and the possibility of any physiological effects of low-dose pesticide exposures in children is a concern. Additional research is needed to determine whether the associations that we observed are evident in other populations. In addition, we emphasize the importance of reducing or eliminating behaviors and conditions that result in pesticide exposures to families of agricultural workers.
